# Robots in Assisted Living Facilities: Scoping Review

**DOI:** 10.2196/42652

**Published:** 2023-03-06

**Authors:** Katie Trainum, Rachel Tunis, Bo Xie, Elliott Hauser

**Affiliations:** 1 School of Nursing The University of Texas at Austin Austin, TX United States; 2 School of Information The University of Texas at Austin Austin, TX United States

**Keywords:** robotics, long-term care, nursing home, residential care, scoping review, review method, robot, aging, elder, older adult, gerontology, geriatric, senior living

## Abstract

**Background:**

Various technological interventions have been proposed and studied to address the growing demand for care of residents in assisted living facilities, in which a preexisting shortage of professional caregivers has been exacerbated by the COVID-19 pandemic. Care robots are one such intervention with the potential to improve both the care of older adults and the work life of their professional caregivers. However, concerns about efficacy, ethics, and best practices in the applications of robotic technologies in care settings remain.

**Objective:**

This scoping review aimed to examine the literature on robots used in assisted living facilities and identify gaps in the literature to guide future research.

**Methods:**

On February 12, 2022, following the PRISMA-ScR (Preferred Reporting Items for Systematic Reviews and Meta-Analyses extension for Scoping Reviews) protocol, we searched PubMed, CINAHL Plus with Full Text, PsycINFO, IEEE Xplore digital library, and ACM Digital Library using predetermined search terms. Publications were included if they were written in English and focused on the use of robotics in assisted living facilities. Publications were excluded if they did not provide peer-reviewed empirical data, focused on user needs, or developed an instrument to study human-robot interaction. The study findings were then summarized, coded, and analyzed using the Patterns, Advances, Gaps, Evidence for practice, and Research recommendations framework.

**Results:**

The final sample included 73 publications from 69 unique studies on the use of robots in assisted living facilities. The findings of studies on older adults were mixed, with some studies suggesting positive impacts of robots, some expressing concerns about robots and barriers to their use, and others being inconclusive. Although many therapeutic benefits of care robots have been identified, methodological limitations have weakened the internal and external validity of the findings of these studies. Few studies (18/69, 26%) considered the context of care: most studies (48/69, 70%) collected data only on recipients of care, 15 studies collected data on staff, and 3 studies collected data on relatives or visitors. Theory-driven, longitudinal, and large sample size study designs were rare. Across the authors’ disciplines, a lack of consistency in methodological quality and reporting makes it difficult to synthesize and assess research on care robotics.

**Conclusions:**

The findings of this study call for more systematic research on the feasibility and efficacy of robots in assisted living facilities. In particular, there is a dearth of research on how robots may change geriatric care and the work environment within assisted living facilities. To maximize the benefits and minimize the consequences for older adults and caregivers, future research will require interdisciplinary collaboration among health sciences, computer science, and engineering as well as agreement on methodological standards.

## Introduction

There is a severe need to provide quality care for the world’s growing population of older adults and to improve the work environments of their professional caregivers. In the United States, the population aged ≥65 years is projected to increase from 49 million in 2016 to 95 million in 2060 [1], and adults in the United States aged >65 years have a 70% chance of eventually requiring some type of long-term care [2]. A study conducted since the start of the COVID-19 pandemic found that 99% of nursing homes and 96% of assisted living facilities in the United States are facing staffing shortages [3]. With >14% of their workforce lost since February 2020, nursing homes have been forced to limit new patient admissions, thus preventing older adults from accessing care [4]. The growing population of older adults, coupled with current caregiver shortages, has led to a severe mismatch between the individuals who need care and those who provide it.

Much effort has been devoted to developing technological interventions to ameliorate the mismatch between care needs and the capacity and quality of care for older adults. An increasing number of robotics and gerotechnology researchers are designing, developing, and evaluating care robots to provide physical assistance and social support to older adults and their caregivers [5]. Countries such as Germany, the United Kingdom, the United States, and Japan have provided economic support to care robotics research [6-8]. Care robots are increasingly highlighted as an innovative way to provide geriatric care, and in a recent European Commission report, 20% of the most influential information technologies for aging projects included care robots [9,10]. Preliminary evidence suggests that care robots have the potential to improve the health of older adults, improve their general well-being and social interactions, and reduce their loneliness [11,12].

Despite these potential benefits, ethical concerns regarding the adoption of robots in aged care remain, including questions about autonomy, deception, and safety [5]. Barriers to the implementation of care robots include technical difficulties, limited capabilities, and negative perceptions [13]. With respect to caregivers, robots have both positive and negative effects on the work environment [14]. Together, these factors present a substantial headwind for both researchers and practitioners as they attempt to develop effective robotic interventions and understand related effects and best practices.

Nevertheless, the implementation of robots in care settings will have profound effects on health care delivery and work environments. As a result of the potential for these wide-ranging effects, several prior literature reviews have focused on care robotics. Existing reviews have examined in-home use of robots to promote aging in place [15-17], specific robotic applications (eg, telepresence robots) [18], relevant ethical issues [5], factors that affect the acceptability or implementation of care robots [13,19], and the impact of robots on caregivers [14]. We aimed to expand upon previous research by focusing our review within the context of assisted living facilities specifically. In addition, our review encompasses all types of robotic applications. Prior literature reviewing research on robots for older adults tends to focus specifically on social robots and on psychological or cognitive outcomes [20-24]. In comparison, our review encompasses a broader picture of robotic research, interventions, and outcomes relevant to caregivers and patients in this setting.

Instead of focusing on quality assessment and synthesis of a well-defined research question, scoping reviews map the current state of knowledge on a topic and identify gaps for future research [25-28]. This form of the review is thus appropriate to our broad research questions: (1) What is known about robots used in assisted living facilities? (2) What research methods, designs, and populations were used in this research? and (3) What gaps exist in the literature and warrant future research?

## Methods

This study adhered to the PRISMA-ScR (Preferred Reporting Items for Systematic Reviews and Meta-Analyses extension for Scoping Reviews) protocol [29]. The PRISMA-ScR checklist is provided in Multimedia Appendix 1 [29].

### Round 1: Keyword Search

As the subject under investigation was interdisciplinary—the use of robots in assisted living facilities—we searched databases in engineering, computer science, and health sciences: PubMed, CINAHL Plus with Full Text, PsycINFO, IEEE Xplore digital library, and ACM Digital Library. On February 12, 2022, we searched PubMed for titles and abstracts using the following sets of terms: (“robot*”) AND (“senior living facilit*” OR “residential facilit*” OR “independent living” OR “assisted living” OR “senior living center*” OR “nursing home*” OR “skilled nursing facilit*” OR “intermediate care facilit*”) AND (“aged” OR “older” OR “elderly”). These search terms were developed from the authors’ previous experience and by examining prior literature reviews in the field. To retrieve a full scope of the literature on our topic of interest, we imposed no time limit on years of publication. Then, we searched CINAHL Plus with Full Text and PsycINFO by titles and abstracts using the same sets of search terms. We then excluded duplicate publications using an electronic screening tool.

In addition, on February 12, 2022, we searched the IEEE Xplore digital library and ACM Digital Library (ACM Full-Text Collection) using the same sets of search terms. We searched IEEE by metadata (titles, abstracts, and indexing terms) and used the 2012 ACM Computing Classification System with the filter “Robotics” to search the ACM Digital Library. Duplicate publications were removed using an electronic screening tool. The publications identified from PubMed, CINAHL, and PsycINFO were combined with the publications from the ACM and IEEE databases, and additional duplicates were removed. A detailed search strategy for each database is provided in Multimedia Appendix 2.

### Round 2: Screening of Titles and Abstracts

Next, the first author (KT) screened each of the nonduplicate papers by title and abstract using predetermined inclusion and exclusion criteria. The results were cross-examined by the other 3 authors, and disagreements were resolved through discussion. Inclusion criteria were as follows: (1) full text written in English and (2) focus on the use of robotics in assisted living facilities. We used the National Library of Medicine’s Medical Subject Headings definition of robotics: “the application of electronic, computerized control systems to mechanical devices designed to perform human functions” [30]. Smart assistive devices (eg, walkers, canes, and transfer devices) and ambient assisted living technologies without a robotic platform were thus excluded from the review. *Assisted living facility* was defined as any residential setting that provides long-term care to older adults, consistent with prior literature reviews [11]. Studies on the in-home use of robots to promote aging in place were excluded. Projects that did not study the robot in a real-world setting were also excluded (eg, those that studied the robot in a laboratory environment or studied the infrastructure behind the robot). Publications were excluded if they (1) did not provide peer-reviewed empirical data (eg, literature reviews, opinion pieces, system architectures, and dissertations), (2) focused on user needs to guide future robot development, or (3) developed an instrument to study human-robot interaction.

### Round 3: Screening of Full Text

The remaining papers were screened by full text using the same predetermined inclusion and exclusion criteria.

### Round 4: Coding and Analysis of Full Text

Data from each of the papers in our final sample were then coded by publication year, study aim, research method, participants’ characteristics, sample size, country and setting where data collection took place, specific robot studied, outcome measures, length of study, and key findings. Two reviewers (KT and RT) completed the coding independently; disagreements were resolved through discussion.

Our data analysis, presentation, and discussion of results follow the PAGER (Patterns, Advances, Gaps, Evidence for practice, and Research recommendations) framework [28], identifying 5 key patterns in the reviewed literature, along with advances, gaps, evidence for practice, and research recommendations for each. Following the PAGER framework [28], the 5 patterns were identified through a patterning chart analysis of the study findings. The patterning chart displays key themes and how they are distributed across publications, which is then used to highlight important patterns and gaps in the included literature (Multimedia Appendix 3 [31-104]).

## Results

### Overview

In this section, we first describe the results of our search and screening process. We then report the key descriptive characteristics of the studies in our final sample. Finally, we describe the 5 key patterns identified from the findings of the final studies.

### Search and Screening Results

During round 1, keyword search, our PubMed search yielded 108 publications. Our CINAHL search yielded 75 publications including 37 duplicates, and PsycINFO search yielded 75 publications including 31 duplicates. Excluding the 68 duplicates, 190 papers remained from the health sciences databases. For the engineering and computer science databases, IEEE Xplore digital library yielded 437 publications and ACM Digital Library yielded 58 publications. Two duplicates were removed from the ACM and IEEE databases, resulting in a total of 493 publications. Combining these with the 190 papers from PubMed, CINAHL, and PsycINFO revealed 4 additional duplicates, resulting in a total of 679 nonduplicate results.

**Figure 1:**
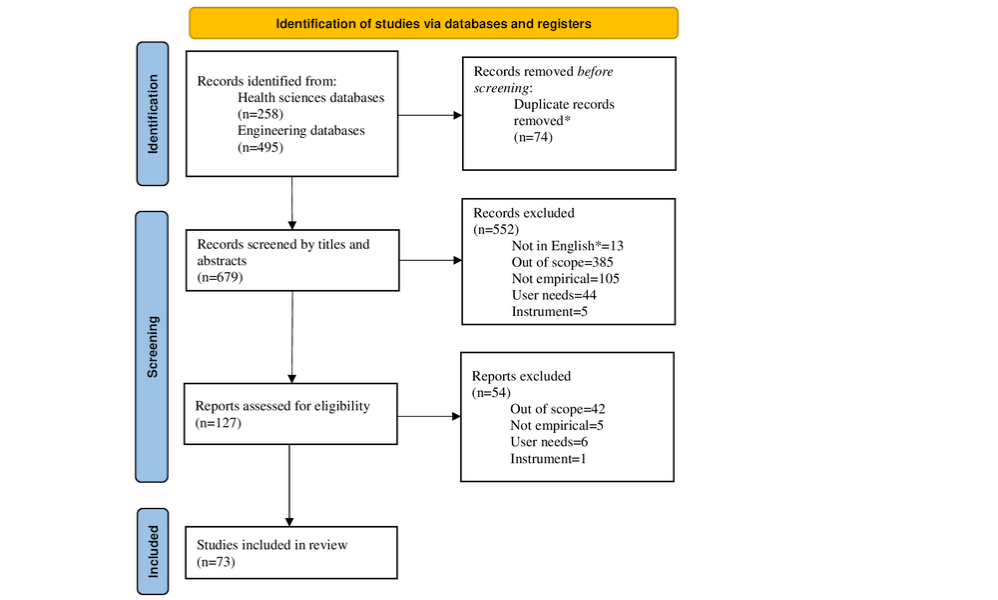
PRISMA (Preferred Reporting Items for Systematic Reviews and Meta-Analyses) flow diagram. *Records that were excluded by automation tools.

During round 2, screening of titles and abstracts, a total of 552 publications were excluded (refer to Figure 1 for details), resulting in 127 publications.

Round 3, screening of full text, resulted in the exclusion of 54 papers. A total of 73 papers remained in the final sample. Figure 1 presents the full search and screening process [105].

### Descriptive Results Based on the Coding of Full Text

The 73 publications included in our final sample reported 69 unique studies; 8 of the publications [31-38] reported on the same 4 studies. Key characteristics of the 73 publications are summarized in Multimedia Appendix 4 [31-104]. The publications were published between 2002 and 2022; >50% (41/73) were published in the last 6 years, suggesting a growing interest in our research topic. Furthermore, 3 studies were inspired by challenges related to the COVID-19 pandemic [39-41]. The studies were conducted in 17 countries—in Asia (22/69, 32%), North America (20/69, 29%), Europe (16/69, 23%), and Oceania (11/69, 16%). On the basis of the authors’ academic disciplines, 33% (23/69) of studies were identified as computer science or engineering oriented, 42% (29/69) were multidisciplinary, and the remaining were from health sciences (15/69, 22%) or social sciences (2/69, 3%).

The included studies were primarily designed to test the feasibility of a robot-based intervention. Feasibility studies assess whether an intervention is relevant and sustainable, and they can include limited efficacy testing [106]. Most of the studies (41/69, 59%) used quasi-experimental designs; the remaining studies were either case studies (21/69, 30%) or randomized controlled trials (7/69, 10%). Overall, 12 studies did not report the study duration; of those that did (57/69, 83%), the majority (37/57) lasted no longer than 6 months. Furthermore, 10 studies lasted <1 week, and 1 study [42] lasted 4 years. Only a few studies (6/69, 9%) [37,42-46] have reported the use of a theoretical or conceptual framework; the majority (63/69, 91%) lacked theoretical guidance. In addition, only a few studies (8/69, 12%) were user informed [47-51] or consulted clinical experts [33,52,53]; the remaining studies (61/69, 88%) lacked users’ or experts’ perspectives.

All studies used convenience samples, ranging from 3 older adults [31,54] or 3 recreational therapists [55] to 245 older adults in the largest study [38]. Five studies did not report the sample sizes [43,47,56-58]. Of the 64 studies that reported the sample sizes, 42 (66%) had no more than 25 participants. Most of the studies (48/64, 75%) collected data only from older adult recipients of care. Overall, 15 studies collected data on staff (eg, caregivers, therapists, board members, management, and preschool staff) [38,48,51,55,59-69], and 3 studies collected data on relatives or visitors [61,64,68]. One study [59], which examined the use of social robots for intergenerational activities, included 30 preschool children, and another study [70] used 6 young adults as a control group. The age of the older adult participants ranged from 55 to 104 years. Of the studies that reported demographic information, all but one [71] included >50% women participants. Of the 69 studies, 27 (39%) reported that they included older adults with cognitive impairments [31,41,42,45,52,53,69,70,72-90].

The studies examined a wide variety of robotic platforms and applications. The most commonly studied robot was PARO (n=22), followed by aibo (n=7) and NAO (n=7). Both PARO and aibo are pet-like robots, whereas NAO is a humanoid robot. The most common application of the robots was to provide social interaction or companionship to older adults (ie, social robots; 60/69, 87%). Less common uses were to assist caregivers with tasks related to their jobs (ie, assistive robots; 6/69, 9%) or to allow relatives and caregivers to provide remote presence to the older adults (ie, telepresence robots; 3/69, 4%).

Outcome measures, both objective and subjective, varied widely across studies. Observations, interviews, and surveys were the 3 most common methods of data collection. Observations were made by the research team, by caregivers employed at facilities, or via the robots’ software. Common observational measures included the number of interactions with robots, time spent interacting with robots, and emotional responses to robots. Observational measures collected by the robots included reaction time [31] and audio and facial tracking [88]. Three studies collected information on medication use [76,79,86]. More than 20 different questionnaires were used, but the most common were the validated and widely used Geriatric Depression Scale (n=4), the face scale mood evaluation (n=3), and the University of California Los Angeles Loneliness Scale (n=3). Furthermore, 4 studies collected physiological data, including blood pressure and heart rate [91], electroencephalogram [70], sleep-wake patterns obtained via wrist actigraphy [83], and salivary chromogranin [92]. Owing to the participants’ cognitive impairments, 3 studies [49,79,86] relied on proxy assessments of quality of life or pain.

### Patterns of Research Findings

In addition to these key characteristics of the studies included in our final sample, we identified 5 key patterns of research findings.

#### Effects, Perceptions, and Experience of Care Robots

The overall findings were mixed; some studies suggested therapeutic effects of the robots, whereas others were neutral or inconclusive. The most commonly reported benefits of the social robots were improved mood and emotional states [33,41,42,44,49,52,55,72,74,76,78,80,81,84,85,87,92-94] and an increase in social interaction between the residents and other human interactors [31,36,41,42,52,55,58,59,63,69,72,75,81,82, 95-98], including caregivers [69] and preschoolers [59]. Other commonly reported benefits of the robots were reduced loneliness [36,92,99], evoked positive memories of pets [59,65,85,96,100], improved quality of life [53,79] and well-being [65], reduced pain and pain medication use [85,86], cognitive stimulation [31,59,78], and improved behavior [49,55,80]. Several robots promoted movement [50,62,72,100,101], and 1 robot prevented unexpected falls [69]. Four studies [45,51,69,71] investigated robots that assist with medication administration, and in 1 study [69], the robot successfully prevented a medication error. Less frequently cited but objectively measured benefits included improved neuroactivity [70], reduced stress [92], decreased blood pressure [91], and improved sleep [83]. Several studies have investigated whether robotic pets could achieve the same benefits as traditional animal-assisted therapy. Three studies found that these robots were able to reduce loneliness [99] and stimulate interaction [75,82], with no differences between the robot and a live dog. One study [89] found that attention toward the robot decreased with time but remained stable with a live dog, whereas another study [35] found that the interaction was statistically significantly greater with the robot than with a live dog.

Residents and caregivers primarily reported positive experiences of using the robots and had positive attitudes toward the robots. However, a few studies [36,41,55,66,68,77,87,96] reported disapproval, and 3 studies [36,65,78] found that human companionship or human-facilitated interventions were preferred by the older adults. In 2 studies, the older adults perceived the robots to be dependent on them, which resulted in a sense of unwanted responsibility [41,96]. In 2 other studies, the older adults were not interested in interacting with the robots because they perceived the robots as toys [36,77]. In 1 study [66], the staff’s perception of the robots’ agency decreased over time.

Our review illuminated several concerns related to care robots as well as barriers to their use. Ethical concerns regarding privacy [54,64,69,95], maintaining autonomy [68,95], and age appropriateness of the robots [36,77] were common themes. One study illuminated safety concerns regarding relatives responding to emergencies via a telepresence robot instead of caregivers [64]. Barriers to use included technical difficulties [38,45,51,54,57,64,77,84,95], difficulty hearing [64,85,88,102] and seeing [48] the robots, and physical limitations [48,51,85,96,100]. However, other studies found the robots easy to use despite cognitive impairments and technological illiteracy [45,57,67,68,102].

#### Factors Influencing the Effects of Care Robots

Several factors have been repeatedly identified as possibly influencing the impact of care robots on older adults living in assisted living facilities. Gender was one such factor, although the results were inconsistent. For example, one study [102] found no differences between men and women participants, but another study found that robot interactions tended to follow socially constructed gender norms: men participants were primarily interested in the robot’s technical functions (“engineer-style” interaction), whereas women participants interacted with the robot as if it were alive [61]. The results were also mixed with respect to how participants’ age and level of cognitive decline affected robot-based interventions’ efficacy. Some studies found better results with younger participants [90] and those with milder cognitive decline [88,90], whereas others found better results with older participants [53] and those with more advanced cognitive decline [53,70,79,84]. Another potential factor that could have influenced the results of the studies was whether the robots spoke the participants’ native languages. In 3 studies, the robots did not speak the participants’ native language or use an appropriate accent, which may have contributed to reduced participation [66] and reduced satisfaction [34] as well as increased staff involvement for translation services [84].

In several studies, the robots’ limited capabilities reduced their efficacy or reduced the older adults’ interest in the robots [45,51,85,86,96,100,101]. For example, 1 robot’s [101] small size contributed to a reduced range of motion during physical therapy sessions. Another robot [45], which assisted older adults with medication administration, required caregiver presence because it lacked the essential capabilities for medication administration such as offering a glass of water. In 2 studies, the older adults wished that the robot had a companion element [51] or was more human like [100].

Novelty effects were a potential factor that may have affected the effects of care robots. Novelty effects are caused by the initial reaction to a new technology, as opposed to the effects of long-term use once the technology is no longer perceived as new [103]. In 5 studies, the initial positive effects of the robots decreased or were no longer significant by the end of the study [80,84,86,89,91]. However, other studies have demonstrated that engagement with and benefits of the robots increased over time [38,42,44,60,62,98,104]. Although many of these studies were not long enough to refute novelty effects, 1 study [98] demonstrated an increase in interactions over a 7-week period; 1 study [62] demonstrated an increased willingness to interact with the robot over 8 weeks; 1 study [38] found that robot use increased from year 1 to year 2; and a 4-year-long study [42] demonstrated significant improvements in emotional, visual, and behavioral engagement from baseline.

#### Robots’ Impact on Caregivers and the Care Environment

Many studies (27/69, 39%) relied on facilitation of robots by researchers, nursing staff, or relatives [35,38,42-45,48, 52,58,59,61,62,67,72,75-77,79-81,83,84,89,90,96,98,101]. Four of these studies [38,52,62,101] suggested one-on-one sessions or groups of <3 to maximize the benefits of robots, but one-on-one sessions were time intensive for caregivers [38]. Of the few studies that compared mediated and nonmediated interventions, 2 studies [43,59] found better results with less staff mediation, and 3 studies [61,72,96] found that the interventions were more effective with staff mediation.

Caregiver shortages have been repeatedly cited as a rationale for studying robots in assisted living facilities, but few studies have addressed the impact of robots on professional caregivers and their work environments. In studies that explored the impact of robots on professional caregivers, robot use was associated with nursing staff’s attitude toward the robot [65], caregivers’ high workload was identified as a barrier to incorporating the robots into care [65,96], and operating the robots was found to be a burden and increased workload for the staff [84,88]. One study [38] reported that caregivers desired more preprogrammed activities to reduce the workload associated with using a care robot, and another study [81] addressed this need by systematizing the use of a recreational robot, significantly increasing participation. Another study presented a system that allowed caregivers to teach a robot how to facilitate a game with residents and allowed caregivers to personalize the robot’s behavior [67]. Several studies emphasized that care robots were not meant to replace nurses, but instead they should be treated as an adjunct method of providing care [43,52,65]. One robot that played games with the residents freed caregivers to perform other tasks [33], caregivers in 1 study appreciated the help of a medication delivery robot [51], and another robot demonstrated the potential to reduce caregiver burden by responding to nurse calls and collecting real-time patient information [69]. The only study to compare job satisfaction before and after a robot intervention found a significant increase for the control group only, which received no robot intervention [66].

#### Comparisons of Robot- and Human-Facilitated Interventions

Instead of comparing robot-facilitated interventions with human-facilitated control groups, most studies (68/69, 99%) either had no control group or compared robot interventions with control groups that received treatment-as-usual. The studies that used treatment-as-usual control groups provided little to no description of usual care or how it was controlled for. This makes it impossible to determine whether the benefits discovered were because of the robot itself or because of the increased attention from being in a research study. Only 1 study in our review directly compared the effects of a robot-facilitated intervention with a comparable human-facilitated intervention [39]. The results of that study indicated that the therapeutic effects of occupational therapist–led sessions were significantly greater than those of robot-directed sessions. The authors concluded that robot-facilitated sessions cannot replace sessions with occupational therapists; however, they suggested that in settings with limited human resources, robots could be an appropriate alternative to occupational therapists [39].

#### Methodological Approaches to Care Robotics in Assisted Living Facilities

Several methodological limitations were noted throughout the final sample of studies included in our review. All studies in this literature review relied on convenience samples, and 5 studies lacked reporting on participant characteristics [43,47,56-58]. Observations, interviews, and surveys were the 3 most common methods of data collection. Observations were made by the research team, by caregivers employed at facilities, or via the robots’ software; however, little to no information was provided on how the assessors were trained. Finally, >20 different questionnaires were used, and there was little discussion about the reliability and validity of these measurement tools.

## Discussion

### Overview

This review examined 73 publications from 69 unique studies on the use of robots in assisted living facilities. The findings of studies on older adults were mixed, with some studies suggesting positive impacts of robots, some expressing concerns about robots and barriers to their use, and others being inconclusive. Although many therapeutic benefits of care robots were identified, methodological limitations weakened the internal and external validity of the findings of the studies. Few studies (18/69, 26%) considered the context of care: most studies (48/69, 70%) collected data only on recipients of care, 15 studies collected data on staff, and 3 studies collected data on relatives or visitors. Theory-driven, longitudinal, and large sample size study designs were rare. Across the authors’ disciplines, a lack of consistency in methodological quality and reporting makes it difficult to synthesize and assess research on care robotics.

Using the PAGER framework [28], we synthesized our findings into five patterns: (1) effects, perceptions, and experiences of care robots; (2) factors influencing the effects of care robots; (3) robots’ impact on caregivers and the care environment; (4) comparisons of robot- and human-facilitated interventions; and (5) methodological approaches to care robotics in assisted living facilities. Table 1 presents an overview of the analysis of these patterns. We discuss the implications of each in detail in the following sections.

**Table 1:** PAGER (Patterns, Advances, Gaps, Evidence for practice, and Research recommendations) framework.

Pattern	Advances	Gaps	Evidence for practice	Research recommendations
1. Effects, perceptions, and experience of care robots	Evidence shows mixed effects (positive, neutral, or inconclusive) and mostly positive perceptions and experiences of robots.	Further systematic research is needed to fully understand the effects, perceptions, and experiences of robot use.	There is growing evidence of the potential of robots to improve the health of older adults living in assisted living facilities.	Continue developing innovative robots to meet the needs of older adults and caregivers. Continue investigating the effects, perceptions, and experiences of robots.
2. Factors influencing the effects of care robots	Evidence suggests that a user’s age, cognitive decline, gender, and culture impact the effect of robots.	Further study is needed to confirm and understand the factors that influence effects of care robots to inform personalization of robot interventions.	To develop effective robots, it is important to consider the factors that influence a person’s attitude and response to robots.	Collaborate with interdisciplinary teams to develop personalized robotic interventions, achieve representative samples, and explore novelty effects.
3. Robots’ impact on caregivers and the care environment	Evidence shows that human mediation affects the efficacy of robots. Evidence also shows that robots can increase workload for caregivers, which is a barrier to use.	Further study is needed on how human mediation affects robot efficacy. Evidence on how robots will be implemented into busy workloads is lacking.	It is crucial for caregivers to be considered in the design of robots.	Study the impact of human mediation on the efficacy of robots. Study how robots impact caregivers. Involve caregivers directly in the design of robot interventions.
4. Comparisons of robot- and human-facilitated interventions	Growing evidence supports the benefits of robot-facilitated interventions compared with treatment-as-usual, but when compared with equivalent human-facilitated interventions, the robot is less effective.	Research that compares robot-facilitated interventions and human-facilitated interventions is limited.	Growing evidence supports the benefits of robot-facilitated interventions, but there is little evidence that robots can provide the same quality of care as a human can.	Future research should carefully consider whether a robot-facilitated intervention is appropriate instead of a human-facilitated alternative. Future robot-based interventions should be designed to support human caregivers.
5. Methodological approaches to care robotics in assisted living facilities	A lack of consistency across disciplines in methodological quality and reporting makes it difficult to synthesize care robotics research and perform quality assessments.	Further systematic research is needed on the use of robots in assisted living facilities, with increased control and quality of reporting.	Methodological limitations reduce internal and external validity, making it difficult to make claims about the efficacy or best practices of care robots.	Develop interdisciplinary guidelines for conducting and reporting high-quality studies. Consult content experts to select appropriate and valid measurement tools. Use theory to guide studies.

### Effects, Perceptions, and Experience of Care Robots

Various robotic platforms were presented in the reviewed studies; social robots were the most common (60/69, 87%), with PARO being the most frequently studied (22/69, 32%). Less commonly studied robots were assistive (6/69, 9%) and telepresence robots (3/69, 4%). The studies’ findings were mixed: some suggested therapeutic effects of the robots, but others were neutral or inconclusive. The most commonly reported benefit of the robots was improved mood and emotional states [33,41,42,44,49,52,55,72,74,76,78,80,81,84,85,87,92-94]. Other commonly identified benefits were increased social interaction [31,36,41,42,52,55,58,59,63,69,72,75,81,82,95-98], reduced loneliness [36,92,99], evocation of positive memories [59,65,85,96,100], cognitive stimulation [31,59,78], improved quality of life and well-being [53,65,79], reduced pain and medication use [85,86], improved behavior [49,55,80], and increased movement [50,62,72,100,101]. Similar findings were identified by other literature reviews [11,12]. In addition, there is some evidence that robotic pets could be a feasible alternative to animal-assisted therapy [35,75,82,99].

For the most part, participants accepted the robots and reported positive experiences of using the robots. The most common concerns related to the adoption of robots in the assisted living facilities were privacy [54,64,69,95], autonomy [68,95], age appropriateness of the robots [36,77], and safety [64]. Common barriers to use included technical difficulties [38,45,51,54, 57,64,77,84,95], hearing and vision impairments [48,64,85,88,102], and physical limitations [48,51,85,96,100]. Similar concerns and barriers were noted in other literature reviews [5,13]. Although there is growing evidence of the potential for robots to improve the health of older adults, further research is needed to systematically explore the efficacy of care robots as well as participants’ perceptions and experiences of their use.

### Factors Influencing the Effects of Care Robots

The impact of age and cognitive decline on the efficacy of care robots remains inconclusive. Some interventions appeared to be better suited for younger participants with milder cognitive decline [88,90], whereas others might be better suited for older participants with more advanced cognitive decline [53,70,79,84]. The impact of gender on the efficacy of care robots was also inconclusive (all but 1 study included in our review had a majority of women participants). The use of convenience samples makes it difficult to gain the perspectives of those who are resistant to care robots. Future studies should include study samples representative of assisted living facilities to fully understand the key factors that influence geriatric robotic care. Further research is also needed to examine whether and how novelty effects might affect residents’ and staff’s responses to care robots. Existing robots tend to have limited functions, which may have contributed to a novelty effect. Prestudy exposure to robots might be an effective way to reduce the impact of novelty effects [95].

These findings also suggest a need to personalize robot-based interventions rather than adopt a one-size-fits-all approach. The nursing discipline has a long tradition of valuing person-centered care [107]. Instead of standardizing care to a whole group of people, person-centered care is holistic, individualized to the unique needs of the person, respectful, and empowering [108]. With a person-centered approach, care robots will be more effective and will better meet the needs of older adults living in assisted living facilities. It is worth noting that 2 studies in this review attempted to personalize robot services, which evoked positive memories and engaged the older adults [34,42].

In addition to personalization based on age, level of cognitive decline, and gender, robots should be tailored to other important factors such as cultural backgrounds. This includes, but is not limited to, using the native language of users. A prior integrative review found that a person’s culture influences their attitudes, engagement, likeability, and perceptions toward a robot [109]. Further studies are required to understand the key factors that influence individuals’ attitudes and responses to care robots. The care robotics field will benefit from partnering with nurse researchers and others in the health sciences discipline who have experience in developing and implementing person-centered care.

### Robots’ Impact on Caregivers and the Care Environment

Caregiver shortages have been repeatedly cited as a rationale for studying robots in assisted living facilities, but few studies have investigated the impact of robots on professional caregivers. Caregiver involvement was essential to the success of many robotic interventions, but few studies considered how robots would be implemented within an already heavy workload. Sharkey [110] argued that the benefits of robots are likely the result of skilled and careful use by caregivers and family members. Our findings support this claim and suggest that human mediation plays an important role in the efficacy of care robots; however, further research is needed to fully understand the impact of robot adoption and use on staff and family members. Knowing whether and how much human mediation is required to achieve the full benefits of care robots is essential because if the use of a robot is burdensome for caregivers, caregiver burnout will worsen or robots will not be used to their full potential.

The few studies in our review that focused on caregivers suggest that robots have the potential to increase the capacity for care by freeing caregivers to perform more meaningful tasks; however, robots also have the potential to increase workload, which is a barrier to their use. One study successfully increased participation by systematizing a robotic program to reduce barriers for caregivers [81]. It is crucial for researchers to carefully consider caregiver needs when designing robots; otherwise, the benefits identified in this literature review will not be achieved. Furthermore, future studies should address how robots will be implemented into an already busy workload.

### Comparisons of Robot- and Human-Facilitated Interventions

Instead of comparing the robots with an equivalent human-facilitated control group, most of the studies (68/69, 99%) included in our review either had no control group or compared the robots with treatment-as-usual. Furthermore, these studies provided little to no description of usual care or how it was controlled for. This makes it impossible to determine whether the benefits discovered were because of the robot itself or because of the increased attention from being in a research study.

In a systematic review on the use of robot-assisted therapy for upper limb recovery after stroke, the authors emphasized that there is no reason to believe that robot-facilitated therapy would have better results than human-facilitated therapy if all other variables were the same [111]. The same is true for care robots in assisted living facilities, as evidenced by the studies that showed better results from human-facilitated interventions or preference for human-facilitated interventions [36,39,65,78,88]. Despite the limitations of using treatment-as-usual control groups, high-quality studies that compare robot-led interventions and usual care can be helpful for determining the benefits of care robots in comparison with the current state in assisted living facilities. With a growing gap between the number of older adults needing care and the number of professional caregivers, it might be unrealistic to expect assisted living facilities to implement additional human-facilitated interventions. Therefore, robots may be a more practical alternative. Either way, it is crucial to carefully consider whether a robot-facilitated intervention is appropriate instead of a human-facilitated intervention. More importantly, it may be necessary for researchers to recognize that robots should not fully replace humans and that robot-based interventions should be designed with the goal of supplementing humans.

### Methodological Approaches to Care Robotics in Assisted Living Facilities

Although this review identified many reported benefits of using care robots, these findings should be interpreted with caution. The research practices and methods currently used in the development and evaluation of robotic systems fall short of those expected by the medical informatics and health technology research communities. Methodological limitations in studies on care robotics have been noted in several other scoping reviews [20-22,112,113]. Establishing standards in research design and in the reporting of study findings is urgently needed for this emerging interdisciplinary work, and it will increase the mutual contribution of caregivers and technologists as the field of robotics moves from the laboratory into its application in care settings.

Many of the studies (15/69, 22%), especially those identified in the engineering databases, lacked reporting on methods and participants’ characteristics. This echoes a similar finding of a literature review that examined the use of artificial intelligence for caregivers of individuals with Alzheimer disease [114]. The lack of consistency across disciplines for what is considered high-quality research makes it difficult to synthesize care robotics research and perform quality assessments. As health care technology continues to advance and as disciplines further merge, it is increasingly important for interdisciplinary criteria to be established for studies and publications.

Observations, interviews, and questionnaires were the 3 most common methods of data collection in the reviewed studies; however, the specifics of these methods varied greatly among studies. More than 20 different questionnaires were used, which makes it difficult to compare the results of the studies, and few studies described the reliability and validity of their measurement tools adequately. Future researchers should consult content experts to ensure that appropriate and valid measurement tools are selected for the setting and population. Studies that relied on researchers’ or caregivers’ observations and assessments (ie, most of the studies) provided little to no information on how assessors were trained, which weakens the internal validity of the findings. Furthermore, serious concerns about bias arise from the widespread use of caregivers, who have prior relationships with residents, to observe and assess the residents. Although proxy measures are often appropriate and necessary for assessing participants with cognitive impairments and can sometimes be an efficient method of longitudinal data collection, no reliability assessments of these measures were conducted, and the studies did not supply information on the training of proxy raters.

The methodological limitations of the reviewed studies reduced their internal and external validity, making it difficult to make claims about clinical efficacy or best practices. To improve the level of evidence, attention should be given to developing interdisciplinary guidelines for conducting and reporting on high-quality studies as well as prioritizing theory-driven research. Although the methods used in these studies are commonly accepted for developing, demonstrating, and assessing novel robotic *functionality*, the responsible and successful application of robotic technology in care settings demands an evolution toward the standards of evidence and validity developed within health research broadly.

### Limitations

Our scoping review had several limitations. First, it is possible that important and relevant studies were missed as only 5 electronic databases were searched. To mitigate this possibility, we chose a broad range of databases representing engineering, computer science, and health sciences. Second, we included only publications with full text written in English; therefore, it is possible that we missed important relevant studies written in other languages. Third, our search terms were not exhaustive, and we may have missed important relevant studies that used different terms for “assisted living facility” or “older adults.” To mitigate this possibility, we reviewed the search terms of prior literature reviews in the field and consulted an information science librarian. Fourth, owing to overlaps between robotic platforms and their uses, we did not further categorize the robots by type. Future work should focus on creating clear definitions of the different categories of care robots to facilitate clearer distinctions and comparisons.

### Conclusions

The implementation of robots in assisted living facilities has profound implications for both older adults and professional caregivers. Care robots have the potential to improve the lives of older adults and the work lives of professional caregivers; however, concerns about their efficacy, ethics, and best practices remain. Despite the prevalence of research on this topic, relatively little work has been conducted with a specific focus on assisted living facilities and determining gaps in understanding how robots impact assisted living facilities. Previous research also overrepresents social robots relative to other types of assistive robots [115] and future research should ensure a more holistic approach going forward.

This scoping review identified 5 patterns of existing research (Table 1). Although existing knowledge, gaps, and recommendations for research vary across patterns, there are commonalities across them. Overall, we found a relative lack of systematic research methods commonly accepted in medical informatics to determine the feasibility and efficacy of robots in assisted living facilities. Research on how robots will change both geriatric care and the work environment of assisted living facilities is lacking, limiting our understanding of how robotics might impact the fuller context of care within which it will operate.

Interdisciplinary collaboration among health sciences, computer science, and engineering as well as agreement on methodological standards will be essential to enable care robotics research to realize its potential benefits and minimize its detriments to older adults and their caregivers. Although many approaches should be investigated, we suggest that formal categorizations of care work are a particularly promising artifact that can be used to strengthen the emerging collaborations that constitute care robotics for older adults and their caregivers.

A more holistic categorization of care interventions would provide a promising vantage point for the interdisciplinary negotiations needed to advance care robotics in a way that augments the skills and knowledge of care workers. The scope of care interventions provided by existing care robotics systems is very narrow, as evidenced in this study and elsewhere [116]. A more encompassing sense of what nurses and other care workers actually *do* could greatly inform the science of care robotics. Could nursing ontologies of interventions and outcomes help care robotics research be more accountable to care professionals and their patients? Could a sociological understanding of how nurses provide care inform the development of robotic technology designed to assist caregivers, rather than patients?

These questions and others must be fully explored so that robotic interventions can be appropriately oriented within the full context of care. Only by understanding patient needs and acknowledging existing care professionals’ knowledge and skills can robots assume a contributing role on care teams.

## Appendix

Multimedia Appendix 1 PRISMA-ScR (Preferred Reporting Items for Systematic Reviews and Meta-Analyses extension for Scoping Reviews) checklist.

Multimedia Appendix 2 Detailed search strategy.

Multimedia Appendix 3 Patterning chart analysis.

Multimedia Appendix 4 Summary of the 73 papers in the final sample.

## Abbreviations

PAGER: Patterns, Advances, Gaps, Evidence for practice, and Research recommendations
PRISMA-ScR: Preferred Reporting Items for Systematic Reviews and Meta-Analyses extension for Scoping Reviews
