# Supplementary material for: Robots in Assisted Living Facilities: Scoping Review
Source: JMIR Aging. 2023 Mar 6;6:e42652. doi: 10.2196/42652 (PMC10028516; doi:10.2196/42652)
Supplement: Multimedia Appendix 2 [file aging_v6i1e42652_app2.docx]

# Multimedia Appendix 2

# Search Strategy

The search terms were developed from the authors’ past experience and by examining prior literature reviews in the field. To retrieve a full scope of the literature on our topic of interest, we imposed no time limit on years of publication. All five of the databases were searched on February 12, 2022.

## PUBMED, CINAHL Plus with Full Text, and PsycINFO:

//Searched by titles and abstracts

1 “robot*”

2 “senior living facilit*” OR “residential facilit*” OR “independent living” OR “assisted living” OR “senior living center*” OR “nursing home*” OR “skilled nursing facilit*” OR “intermediate care facilit*”

3 “aged” OR “older” OR “elderly”

4 1 AND 2 AND 3

## IEEE Xplore Digital Library:

//Searched by metadata (titles, abstracts, and indexing terms)

“robot*”

AND “senior living facilit*” OR “residential facilit*” OR “independent living” OR “assisted living” OR “senior living center*” OR “nursing home*” OR “skilled nursing facilit*” OR “intermediate care facilit*”

AND “aged” OR “older” OR “elderly”

## ACM Digital Library (ACM Full-Text Collection):

//Searched using the 2012 ACM Computing Classification System’s filter “Robotics”

(“robot*”) AND (“senior living facilit*” OR “residential facilit*” OR “independent living” OR “assisted living” OR “senior living center*” OR “nursing home*” OR “skilled nursing facilit*” OR “intermediate care facilit*”) AND (“aged” OR “older” OR “elderly”)
