# Supplementary material for: Robots in Assisted Living Facilities: Scoping Review
Source: JMIR Aging. 2023 Mar 6;6:e42652. doi: 10.2196/42652 (PMC10028516; doi:10.2196/42652)
Supplement: Multimedia Appendix 4 [file aging_v6i1e42652_app4.docx]

Multimedia Appendix 4

Summary of the 73 Papers in the Final Sample

| **Author** | **Year** | **Study Aim** | **Method** | **Participants** | **Country** | **Setting** | **Robot Name** | **Outcome Measurement** | **Length of Study** | **Key Findings** |
| --- | --- | --- | --- | --- | --- | --- | --- | --- | --- | --- |
| Montemerlo et al. | 2002 | To assess the feasibility of using a robot to escort elderly | Case study | Not specified | USA | Assisted living facility | Pearl | Observation; interview | 5 days | Poor speech recognition and difficulty adapting velocity to people's walking pace; elderly excited about robot; everyone able to operate robot in 5 minutes; successfully autonomously guided residents |
| Kanamori et al. | 2003 | To determine the usefulness of a pet-type robot for enhancing quality of life | Quasi-experimental | 5 females (mean age: 68.2); 5 control participants (4 female) (mean age: 72.8) | Japan | Nursing home | AIBO | Salivary chromogranin; observation; survey: Ando, Osada & Kodama Loneliness scale, SF-36 | 7 weeks | Reduced loneliness; role-emotional significantly improved; decreased salivary chromogranin (reduced stress) |
| Libin & Cohen-Mansfield | 2004 | Pilot study to compare a robotic cat and a plush toy cat as an intervention for dementia | Quasi-experimental | 9 female residents (age range: 83-98) | USA | Nursing home | NeCoRo | Observation: Agitated Behaviors Mapping Instrument, Lawton's Modified Behavior Steam | 2 days | Decreased agitation with plush; increased pleasure and interest with robotic; more cognitive impairment associated with less engagement with both cats; younger adults paid more attention to robotic; researcher modeled interaction |
| Shibata et al. | 2004 | To study the psychological and social effects of a robot | Quasi-experimental | 23 participants without dementia | Japan | Health service facility | PARO | Observation; survey: face scale, Profile of Mood States | 3 weeks | Moods improved with both PARO and placebo PARO (only simple motions) |
| Kidd et al. | 2006 | To compare a placebo and interactive robot on generated social activity | Quasi- experimental | “about 23 patients” (exact sample size and demographics not reported) | USA | 2 nursing homes | PARO | Observation; survey; interview | 4 months | Study in nursing home B was discontinued because understaffed; robot increased social interactions (especially with caregiver participation), evoked memories of pets; barriers: mismatched expectations, hesitancy toward wild animal, heavy; collective sharing and ability to turn off reduced unwanted sense of responsibility from robot’s perceived dependence |
| Hamada et al. | 2006 | To study the usefulness of a robot therapy with and without intervention | Quasi-experimental | 5 elderly persons with dementia | Japan | Nursing home | Pet-type | Survey: staff’s perception of resident's changes | Not specified | Movement, laughter, expression, and communication increased after therapy; therapy with intervention more effective than without for generating spontaneous reaction; intervener believed to have a significant role in robot therapy |
| Giusti & Marti | 2006 | To observe human robot interaction | Case study | 5 participants (4 female) (age range: 56-85) | Italy | Nursing home: A (severe cognitive/behavioral impairment), B (early-stage dementia) | PARO | Observation; duration of speech | 1 month | Participants remained focused for the whole session; group A interpreted robot as an agent, group B interpreted robot as both an agent and an inanimate object |
| Hamada et al. | 2008 | To study the effect of robot therapy recreation | Quasi-experimental | 11 elderly with dementia | Japan | Nursing home | AIBO | Observation | 5 days | Promising effects on memory, emotion control, and accommodation to society |
| Banks et al. | 2008 | To compare a dog, AIBO, and no animal-assisted therapy at treating loneliness | RCT | 38 residents | USA | 3 long-term care facilities | AIBO | Observation; survey: modified Lexington Attachment to Pets Scale, UCLA Loneliness Scale | 8 weeks | Those who received animal-assisted therapy from robot or dog were less lonely than those who did not; high levels of attachment to both dog and AIBO; no differences between dog and AIBO |
| Tapus et al. | 2009 | To study the use of a socially assistive robot for music therapy compared to a computer simulated robot | Quasi-experimental | 3 older adults (age: 70+) with cognitive impairments | USA | Senior living care facility | Custom humanoid torso mounted on Pioneer mobile platform | Survey: Standardized Mini-Mental State Examination; reaction time | 8 months | No adverse response; participants enjoyed interacting with robot; preferred robot to simulated version; robot led to better results in social interaction, reaction time, and task performance compared with simulation; slight improvement in mental state |
| Tapus et al. | 2009 | ----------- Same as above ----------- | | | | | | | | |
| Kramer et al. | 2009 | To compare the effects of visitation by a person alone, a person with a dog, and a person with AIBO on social interaction | Quasi-experimental | 18 female residents with dementia | USA | Nursing home | AIBO | Observation | 3 weeks | Visitor present and prompted conversation in each scenario; all situations stimulated social behavior, but live dog and AIBO stimulated more than visitor alone; it was suggested that AIBO can stimulate social interaction |
| Kimura et al. | 2010 | To estimate the effect of robot assisted activity on neuroactivity; to compare autonomous and remote controlled AIBO | Quasi- experimental | 11 older adults (age range: 60–97), 6 young adults | Japan | Nursing home | AIBO | Electroencephalography | 1 session | With autonomous robot, all participants improved neuroactivity; remote controlled led to larger improvement in 2 participants; larger improvement in participants with greater cognitive impairment; reduced neuroactivity in half the young adults (lost interest) |
| Tiwari et al. | 2011 | To study the usability, feasibility, and appropriateness of a robot for helping older adults with medications | Case study | 10 participants (age range: 69–94) | New Zealand | Aged care facility | Healthbot robot | Observation (via video recording by robot); survey; interview | 1 session | Well received, easy to use; researcher’s presence was essential to study; mild cognitive impairment and unfamiliarity with technology did not impede usability; barriers: technical errors, limited capabilities; mixed opinions on regular use; unanimous dislike of a more human voice; “five rights” of medication administration |
| Hansen et al. | 2012 | To observe the acceptance and game play patterns of a rehab robot | Quasi-experimental | 15 older adults with mobility problems (age range: 65–95); 10 persons from rehab center | Denmark | Nursing home; rehabilitation center | FESTO | Observation; interview | 1 session | Robot game encouraged older adults to move despite disabilities or pain; wished robot was more human-like and had more features; evoked memories of pets; participants who require more assistive devices resulted in more passive behavior |
| Park et al. | 2012 | To compare residents’ and staff’s satisfaction and usability evaluation of a robot | Quasi-experimental | 17 participants (76% female) (age range: 67–88) | Korea | Nursing homes | Cafero | Survey | 5 weeks | Residents and staff were satisfied with robot, no difference between groups |
| McColl et al. | 2013 | To observe potential user interactions with robot | Case study | 40 older adults (age range: 57–100) (64% female) | Canada | Long-term care facility | Brian 2.1 | Observation: duration of interaction, engagement, compliance; user-acceptance survey | 2 days | Majority engaged and complied with robot; positive attitude and experience; no difference between male and female residents; easy to use, regardless of computer experience; memory game was favorite activity, robot’s ability to display emotions was most-liked characteristic; barrier: hearing loss |
| Recio et al. | 2013 | To compare the effects of modeling movements by a physiotherapist, a physiotherapist and virtual NAO, and a physiotherapist and NAO | Quasi-experimental | 13 inpatients | Spain | Assisted living facility | Nao | Observation | Not specified | Robot developed to reduce the emotional attachment with physiotherapist that makes rehab challenging; each scenario requires one-on-one session with physiotherapist; participants mimicked NAO, which improved technical quality but reduced range of motion; better results with robot than with virtual NAO |
| Chang et al. | 2013 | To study the effects of using PARO for a sensory therapy activity | Quasi-experimental | 10 older adults (age: >65); 2 therapists | USA | Nursing home | PARO | Observation; interview | 8 weeks | Increased physical activity; increased willingness to interact with PARO over time; therapists suggest one-on-one or 2–3 person groups |
| Khosla et al. | 2013 | To study the effect of Matilda on emotional well being | Quasi-experimental | 70 residents | Australia | 3 residential care facilities | Matilda | Observation; survey; interview | 3 days | Residents enjoyed interacting with the robot and felt relaxed talking to it; preferred playing games with the robot, which freed caregiver to perform other tasks; personalized care; dissatisfied with accent |
| Khosla et al. | 2013 | ----------- Same as above ----------- | | | | | | | | |
| Wada et al. | 2013 | To study the effectiveness of robot therapy for persons with dementia and the ability to reduce care burden | Quasi-experimental | 80 participants (89% female) (mean age: 86.2) | Japan | 9 residential facilities | PARO | Observation; survey | 2–5 months | 54% liked PARO, 7% disliked; 23 participants showed positive changes (improved anxiety, irritation, aggression, and depression); no results on care burden provided |
| Robinson et al. | 2013 | To study the psychosocial effects of PARO | RCT | 40 residents (67.5% female) (age range: 55–100) | New Zealand | Rest home; hospital | PARO | Observation; survey: UCLA Loneliness Scale, Geriatric Depression Scale, Quality of Life for Alzheimer’s Disease | 12 weeks | Decreased loneliness in robot group only; touched and talked to/about robot more often than dog; staff started conversation more with robot; more social interaction with robot |
| Robinson et al. | 2016 | Provides additional information on methodology, residents' interactions, and staff perspectives |  | 20 residents from PARO group (70% women) (mean age: 84.4) | ----------- Same as above ----------- | | | | | Residents interpreted PARO as an agent, but were aware it was an artificial object; PARO not as popular as other activities; primary benefit was social facilitation; 6/20 refused to play with robot (because it’s like a toy); decrease in loneliness due to increased social interaction |
| Bäck et al. | 2013 | To examine the use of a robot as an exercise trainer | Case study | 34 residents, 13 staff members | Finland | 3 private nursing homes | NAO H25 | Interview | Not specified | Mostly positive feedback; staff support necessary, especially with passive residents; robot’s small size and coloring made it difficult to see; future studies should investigate long-term impact and compare with human therapists |
| Šabanović et al. | 2013 | To evaluate the use of PARO for multi-sensory behavioral therapy | Quasi-experimental | 7 elderly residents | USA | Nursing home | PARO | Observation | 7 weeks | Interpretive flexibility of robot; mediated by therapist; frequency and length of interactions increased over time; main effect = promoting human social interaction |
| Ahn et al. | 2015 | To analyze the use of entertainment services deployed on a robot | Case study | Not specified | New Zealand | Selwyn Village (hospitals, rest homes, private apartments) | Charlie (with installed entertainment services) | Robot’s software records | Few days | Entertainment services used more often in private spaces; music video service used most often |
| Sung et al. | 2015 | To analyze the effect of PARO-assisted therapy | Quasi-experimental | 12 older adults (mean age: 77.25) | Taiwan | Residential care facility | PARO | Observation: Assessment of Communication and Interaction Skills; Activity Participation Scale | 4 weeks | Communication/interaction skills and activity participation improved |
| Chang & Šabanović | 2015 | To study how different social actors understand and use PARO | Case study | “average 49” participants per session (19% residents, 55% staff, 26% visitors) (majority female) | USA | Nursing home | PARO | Observation; interview | 13 weeks | Majority ignored robot; indirect interaction preferred (if direct, usually nonphysical); interactions reflected own needs/experiences and typical gender roles; mediation by visitors, staff, or other residents had positive effect on residents’ likelihood to interact; interpretative flexibility allowed residents to develop new ways to use the robot; exposure to robot caused perceptions to change and practices to shift related to robot |
| Pfadenhauer & Dukat | 2015 | To study how caregivers use PARO as an activation therapy for nursing home residents | Case study | Not specified | Germany | Church-funded elderly residential care center | PARO | Observation | 1 year | Care worker’s method of holding robot was important for engaging residents; when care workers were an observer (vs. a participant), interaction with the robot was more meaningful and promotes independence; need for research on how robots will change nursing homes; tension between nursing and professional caregiving |
| Robinson et al. | 2015 | To study the effect of a robot on blood pressure and heart rate | Quasi-experimental | 17 residents | New Zealand | Rest home; hospital | PARO | Blood pressure and heart rate at 3 time points | 12 weeks | Systolic and diastolic decreased; diastolic increased with PARO removed; only significant when residents who didn’t interact with robot removed |
| Jøranson et al. | 2015 | To study the effect of a robot-assisted group activity on agitation and depression | RCT | 60 residents with dementia (age range: 62–95) (67% female) | Norway | 10 nursing homes | PARO | Observation: Brief Agitation Rating Scale, Cornell Scale for Symptoms of Depression in Dementia; medication log | 6 months | Improvements in agitation and depression from baseline to 3 months postintervention; facilitated by staff |
| Naganuma et al. | 2015 | To study the effect of AIBO to facilitate self-reliance | Case study | Not specified | Japan | Nursing home | AIBO | Observation | Not specified | Facilitated activity; robot promoted social interaction between residents |
| Piezzo & Suzuki | 2016 | To analyze the feasibility of using Pepper to motivate elderly persons to walk more | Quasi-experimental | 4 older people with a temporary physical and/or mobility impairment (age range: 83–92) | Japan | Nursing home | Pepper | Observation; survey: modified Intrinsic Motivation Inventory | Not specified | All participants chose to follow robot from behind (unlike how elderly walk with their caregivers); not nervous (able to choose distance from robot); very good results on relatedness/trust subscale, acceptable results on enjoyment/interest and value/usefulness subscales |
| Moyle et al. | 2016 | Pilot study on the effect of using a companion robot on engagement and emotional states | Quasi-experimental | 5 female residents with dementia | Australia | Nursing home | CuDDler | Observation: Cohen-Mansfield agitation inventory, Observed Emotions Rating Scale; interview | 5 weeks | Individual sessions facilitated by RN; not all participants accepted robot; technical difficulties and time-consuming; participants believed robot was a toy, not age appropriate; inconclusive effect on agitation |
| Broadbent et al. | 2016 | To investigate the benefits and risks associated with robots | Quasi-experimental | 53 staff; 52 residents (age range: 66–97) (77% women) | New Zealand | Rest home; nursing home | Guide; Cafero | Interview; survey: Geriatric Depression Scale, Quality of Life for Alzheimer’s Disease, Abbreviated Mental Test Score, Short Form 12, Psychiatric Epidemiology Research Instrument, Robot Attitude Scale | 12 weeks | Increased job satisfaction in control group (no robot exposure); English second language for many staff members but study conducted in English; low usage of robots; staff’s perception of the robots’ agency decreased with time |
| Thodberg et al. | 2016 | To compare the immediate behavioral response to a visit from a person with a dog, PARO, or toy cat | RCT | 100 residents (age range: 67.5–93) | Denmark | 4 nursing homes | PARO | Observation | 6 weeks | Dog and PARO triggered the most interaction, but attention toward robot decreased over time (remained constant with dog); those with higher cognitive impairments interacted with animal more than human |
| Jøranson et al. | 2016 | To study the effect of a robot-assisted group activity on quality of life | RCT | 53 participants | Norway | 10 nursing homes | PARO | Observation: Clinical Dementia Rating scale, Quality of Life in Late-Stage Dementia scale; medication usage | 7 months | Positive effect on QOL only for those with severe dementia; decreased psychotropic medication usage for intervention group; inclusion criterion was interest in PARO; facilitated by nursing staff |
| Birks et al. | 2016 | To study recreational therapists’ perception of PARO | Case study | 3 recreational therapists | Australia | Aged care facility | PARO | Interview | 4 months | Not effective for everyone; emotional, behavioral, and social benefits; therapists were hopeful about PARO’s potential; some residents, relatives, and staff had negative experiences |
| Abdollahi et al. | 2017 | To assess the use of Companionbot for improving quality of life | Quasi-experimental | 6 older adults (age range: 63–86) (5 female) with dementia and depression | USA | Senior community | Ryan Companionbot (customized to each participant) | Survey; observation: number of dialogues, time spent interacting with robot | 4–6 weeks | 5/6 requested the robot remain in their room longer; interest did not decline with time; participants enjoyed interacting with robot, but did not feel it could replace human companionship; robot helped maintain schedule, improved mood, and provided mental stimulation |
| Kort & Huisman | 2017 | To explore the use and acceptance of ZORA robot by professionals and residents (1st year of study) | Case study | 14 board members; 1 management staff; 17 professionals; unspecified number of residents | Netherlands | 15 nursing homes (14 organizations) | Nao (Zora installed) | Observation; interview; survey: modified Usefulness, Satisfaction, Ease of Use | Multi-year | Goal was to gain experience using care technology and prepare for future; barrier: Wi-Fi; desire for collaboration with other organizations; professionals have positive opinion of robot |
| Huisman & Kort | 2019 | To evaluate whether Zora can be extended to more groups and types of clients (reports on 1st and 2nd year of study) |  | 14 board members; 1 management staff; 17–19 professionals; 245 clients | ----------- Same as above ----------- | | | | | Zora was widely used, increased use in 2nd year; all organizations planned to continue using as an alternative activity; positive effect on clients and staff; barriers: start-up time, software failures (decreased Usefulness, Satisfaction, Ease of Use scores in 2nd year); team meetings were a facilitator; one-to-one situations effective, but time-intensive for caregivers; professionals desired preprogrammed activities to simplify |
| Thinh et al. | 2017 | To evaluate the feasibility of a self-feeding system | Case study | Not specified | Vietnam | Nursing home | Self-feeding robot | Not specified | Not specified | Positive feedback |
| Aaltonen et al. | 2017 | To study the usage of a telepresence robot | Case study | 1 resident (age: 82), 2 daughters, care staff | Finland | Nursing home | Double | Observation; interview | 10 weeks | Positive experience; resident preferred phone conversation (possibly due to hearing issues); potentially more beneficial for residents who live alone or have infrequent visitors; barrier: network connectivity; privacy concerns, safety concerns |
| Khosla et al. | 2017 | To study engagement and acceptability of a robot | Quasi-experimental | 115 persons with dementia (age range: 65–90) | Australia | 4 residential aged care facilities | Matilda | Observation; survey | 4 years | Improved emotional engagement, visual engagement, and behavioral engagement; positive attitudes toward Matilda; high ratings on usefulness and enjoyment; demonstration by researchers; personalization of robot services |
| Rantanen et al. | 2017 | To study the safety and usability of a telecare robot to promote medication adherence | Quasi-experimental | 17 patients (mean age: 73) (65% male) | Finland | Nursing home | Evondos E300 & Evondos Telecare system | Observation | 457 days | No medication doses missed |
| Nihei et al. | 2017 | To examine whether a robot can increase communication among mildly demented elderly | Quasi-experimental | 9 residents (age range: 80–90) (1 male) | Japan | Group homes for mildly demented elderly | Teddy bear social robot (Fujitsu Labs) | Observation | 4 days | Communication between tenants and robot increased; time spent with robot increased |
| Koh & Kang | 2018 | To study the effect of PARO on cognition, emotion, problem behavior, and social interaction | Quasi-experimental | 33 elderly (age: >65) with dementia | Korea | Long-term care facility | PARO | Observation; survey: Korean Mini-Mental State Examination, Apparent Emotion Rating Instrument, Korean Cohen-Mansfield Agitation Inventory | 6 weeks | Larger improvements in positive emotions and problem behaviors in experimental group compared with control; improved social interaction; no significant difference in cognitive function; facilitated activity; groups consisted of 3 residents to maximize effects |
| Joshi & Šabanović | 2019 | To explore the use of social robots for intergenerational activities | Quasi-experimental | 28 older adults (age: 55+), 30 preschool children, 5 preschool staff, 7 assisted living staff | USA | Combined assisted living/dementia care and preschool facility | PARO, Joy For All, Nao, Cozmo | Observation: inter-generational observation scale (IOS), number of interactors | 4 months | Pet-like robots promoted spatial proximity, provided cognitive stimulation, and invoked memories; toy-like robots overwhelming; Joy For All least appealing because not realistic; breaks promoted interaction; more interaction without staff mediation |
| Ostrowski et al. | 2019 | To explore how a social robot shapes older adults’ interactions | Quasi-experimental | 19 older adults (age range: 67–99) (68% female) | USA | Assisted-living community | Jibo | Survey: Inclusion of Community in Self Scale; observation: number of people in common space; interview | 3 weeks | More people in common space and higher community connectedness after 3 weeks; potential to stimulate interpersonal interactions; concerns about privacy, autonomy, technological difficulties, and imagining the robot in daily life; initial level of openness toward robot was correlated with openness at end of study; support for group-level intervention and prestudy exposure to robot to reduce novelty effects |
| Greer et al. | 2019 | To study the benefits of a social robot intervention | Quasi-experimental | 8 participants | USA | Senior living facility | NAO | Survey: Face Scale, UCLA Loneliness Scale, Geriatric Depression Scale; interview; observation | 3 weeks | Decreased depression; engagement with robot increased after 2nd and 3rd session; no significant changes in mood or loneliness |
| Motahar et al. | 2019 | To develop a low-cost assistive robot and gain understanding of end users’ perception | Case study | 20 older-adults (70% female) (age range: 78–101); 8 caretakers | Bangladesh | Older adult care center | SHEBA | Interview; survey | Not specified | High ratings on utility, usability, satisfaction; barriers: physical impairments, technology illiteracy; medication delivery perceived as helpful by older adults and caregivers; wish that robot had companion element |
| Dino et al. | 2019 | To evaluate the effectiveness and user satisfaction of robot-based, internet-delivered cognitive behavioral therapy | Quasi-experimental | 4 participants (age: 60+) with depression | USA | Senior living facility | Ryan Companionbot | Observation; survey: Patient Health Questionnaire-9 Item, Geriatric Depression Scale, Saint Louis University Mental Status Examination, Face Scale | 4 weeks | Inconclusive results; average sentence length increased (suggests increased comfort with robot or decreased depression symptoms); user satisfaction |
| Yamazaki et al. | 2019 | To study the effect of robotic mediation in promoting conversation to improve behavioral and psychological symptoms of dementia | Quasi-experimental | 5 females with dementia (mean age: 87.8) | Japan | Long-term residential care facility | Telenoid | Neuropsychiatric Inventory Nursing Home Version | 10 weeks | Decrease in total score and occupational disruptiveness after 5 weeks (not significant at 10 weeks); decrease in anxiety and appetite/eating change at 5 weeks; operator of robot was familiar with participants’ life histories |
| Niemelä et al. | 2021 | To study how a telepresence robot impacts residents, family, and care workers; to identify barriers to adoption | Case study | 3 residents | Finland | 2 residential care homes | Double | Observation; interview | 6–12 weeks | Technical difficulties; residents and family members reported increased feeling of presence; residents preferred phone; family members and care workers concerned about privacy and ethics; care workers’ experience mostly positive |
| Itai et al. | 2020 | To compare scenario-type and non-scenario-type robot recreation programs (to reduce barriers for staff by systematizing the robotic program) | Quasi-experimental | 10–15 elderly with dementia (nursing home);  6 people (dementia group home) | Japan | Special nursing home; dementia group home | Yumeru/Neruru; AIBO | Observation: participation, frequency of communication, simplified Affect Rating Scale | Not specified | Participation higher in scenario-type; communication 10x more frequent and positive emotions higher in robot activity vs. normal life (eating in common space); positive correlation between communication frequency and Affect Rating Scale score |
| Pou-Prom et al. | 2020 | To study the feasibility and compare the effect of using a conversational robot (remote controlled and autonomous) vs. a human for a cognitive assessment task | Case study | 19 participants with Alzheimer's (84% female) (age: 55+) | Canada | Nursing home | Milo R25 robot from Robokind | Observation; survey; audio and facial tracking data | 1 year | Conversations with human were smoother and richer; better results with those who have milder cognitive decline; technical limitations made autonomous robot hard to understand; robot well liked and captured interest; demonstrated feasibility of using conversational robot for cognitive monitoring; controlling robot was a burden for operator |
| Kolstad et al. | 2020 | To study current use of robots in nursing homes | Case study | Facility managers; nursing staff; residents (sample size not reported) | Japan | 3 nursing homes | PARO, Pepper, Qoobo | Observation; interview | Not specified | Evoked memories; use of PARO depends on nursing staff attitude; staff’s busy schedule left little time for PARO; not considered better than humans, but valuable for assisting and relieving work overload; positive impact on older adults’ well-being |
| Tummers et al. | 2020 | To compare immediate level of engagement toward a robot, a dog, or a human | Quasi-experimental | 12 people with dementia | Netherlands | Care home | Pleo | Observation; interview | Not specified | More conversation with dog or robot present, but no clear preference; robot acted as social stimulant; residents formed strong emotional attachments toward robot |
| Louie & Nejat | 2020 | To study the effectiveness and caregivers’ experience using a learning from demonstration system for a socially assistive robot and to study the residents’ experience | Case study | 5 caregivers;  18 residents (88% female) (age range: 73–93) | Canada | Residential care facility | Tangy | Survey: NASA task load index, System Usability Scale | Not specified | Caregivers found system easy to use, moderately low workload, and were able to successfully teach robot; residents enjoyed robot and found it helpful and entertaining |
| Salichs et al. | 2020 | To study how users perceive a robot | Case study | 10 elders; 7 caregivers; 3 relatives | Spain | Nursing home | Mini | Survey | 2 months | Elders believed robot was useful, easy to use, and motivational, but didn’t understand how it could help users gain autonomy or reduce caregiver demands; positive attitude toward robot; robot perceived as a machine; caregivers rated usability higher than elders did |
| Obayashi et al. | 2020 | To study the impact of age, gender, and stage of dementia on the effects of a communication robot intervention | Quasi-experimental | 78 residents (87% female) (mean age: 86.5) | Japan | 4 nursing homes; 2 rehabilitation facilities | Cota & Parlo | Observation: Degree of Daily Life Independence Score for People with Dementia | 8 weeks | Participants 80 years old or older and those with more advanced dementia had better results; more improvement in quality of life for experimental group |
| Obayashi & Masuyama | 2020 | To study the effects of a communicative robot within a sensing system to determine best implementation strategies | Quasi-experimental | 4 care workers; 2 elderly women with mild dementia (87 and 104 years old) | Japan | Nursing home | Sota | Survey: Profile of Mood States; observation | 3 months | Frequent nurse calls found to enhance psychological burden; robotic sensors reduced caregiver burden by proving real-time information about patients; conversations with robot prevented a medication error and unexpected falls; conversations between care workers and residents increased; conversations with robot increased; caregivers gradually accepted robot as assistants; privacy concerns |
| Fan et al. | 2021 | To evaluate the effect of a socially assistive robot on activity and social engagement | Quasi-experimental | 9 older adults (retirement); 6 older adults (senior living); staff (sample size not reported) | USA | Retirement community; senior living center | Ro-Tri | Robot software; survey: Robot Acceptance Survey (RAS), visual analog scale (VAS) | 3 weeks | Older adults and staff had positive perceptions (more positive after study); older adults remained engaged; visual attention toward robot and partners increased; interaction effort increased |
| Jøranson et al. | 2021 | To study the effects of a PARO group activity on sleep patterns | RCT | 54 participants with dementia (age range: 62–95) (65.4% female) | Norway | 10 nursing homes | PARO | Sleep wake patterns via wrist actigraphy | 12 weeks | Robot had positive effect on sleep; facilitated by nurses |
| Obayashi et al. | 2022 | To compare the effects of a soft-type robot with and without communicative functions | Quasi-experimental | 30 residents (mean age: 86) | Japan | Special nursing home for older people | Mon-chan | Observation: Video Coding Protocol Incorporating Observed Emotion; survey: interRAI | 4 weeks | Mood and behavior improved regardless of communicative ability |
| Liao et al. | 2021 | To compare the effects of a singing group led by social robots and occupational therapists during the COVID-19 outbreak | Quasi-experimental | 14 residents (age: 65+) | Taiwan | Senior care center | Zenbo | Survey | 8 weeks | Sessions led by occupational therapist had greater therapeutic effects than did robot-directed sessions |
| Tulsulkar et al. | 2021 | To study the effectiveness of human–robot interactions | Quasi-experimental | 14 cognitively impaired residents | Singapore | Nursing home | Nadine | Computer vision techniques; observation: Observed Emotion Rating Scale, Menorah Park Engagement Scale | 2 weeks | Residents engaged positively; positive change in emotional state and engagement level; robot’s presence required increased staff activity; need for a better speech recognizer; improved communication and mental processes for those with cognitive impairment; residents’ expressions became more neutral over time |
| Follmann et al. | 2021 | To determine the acceptance and benefits of using a robot for virtual encounters during COVID-19 | Quasi-experimental | 70 participants (mean age: 83) (73% female) | Germany | 2 nursing homes; hospital | Home care robot temi | Observation; survey; interview | 2 months | No effect on loneliness; positive attitudes toward robot |
| Van Orden et al. | 2022 | To describe a robotic pet distribution program during the COVID-19 pandemic | Case study | 13 veterans (age range: 71–102) | USA | VA dementia care residence | Joy For All companion pets | Interviews with care staff | Not specified | Staff reported reduced anxiety and increased social interactions; challenge when residents believed robots were real; demand exceeded supply |
| Lin et al. | 2022 | To study whether a socially assistive robot promotes human–human interaction and to study whether type of activity influences human–human or human–robot interaction | Quasi-experimental | 14 residents (age range: 70–90) (79% women) | USA | 2 assisted living facilities | NAO | Observation | 3 weeks | Robot-mediated activities promoted human–human and human–robot interaction; type of activity influenced type and result of interaction (desired engagement should be determined before designing robot) |
| Pu, Moyle, & Jones | 2020 | To study how persons with dementia and chronic pain perceive a robot | Case study | 11 participants with dementia and pain (age range: 65–94) (81.82% female) | Australia | 3 residential care facilities | PARO | Interview | 6 weeks | Positive attitudes; positive effect on mood and pain; evoked memories of pets; those with more cognitive impairment perceived robot as real animal, while those with less impairment perceived it as toy; barriers: heavy, difficult to understand, and limited capabilities |
| Pu, Moyle, Jones, & Todorovic | 2020 | To study the effect of PARO on pain and behavioral/psychological symptoms of dementia and chronic pain | RCT | 42 residents with dementia and chronic pain (age range: 65–97) (85.7% female) | Australia | 3 long-term care facilities | PARO | Observation: Pain Assessment in Advanced Dementia, nurse estimated pain, Cohen-Mansfield Agitation Inventory, Cornell Scale for Depression in Dementia, Rating Anxiety in Dementia scale; medication usage | 6 weeks | Less observed pain and pain medication usage for robot group (vs. usual care), but results converged at week 6 (possibly due to limited behavioral repertoires); not facilitated |

Note: RCT, randomized controlled trial
